# Cortical responses to changes in acoustic regularity are differentially modulated by attentional load

**DOI:** 10.1016/j.neuroimage.2011.09.006

**Published:** 2012-01-16

**Authors:** Maria Chait, Christian C. Ruff, Timothy D. Griffiths, David McAlpine

**Affiliations:** a Ear Institute, University College London, London, UK; b Wellcome Trust Centre for Neuroimaging, University College London, London, UK; c Institute of Cognitive Neuroscience, University College London, London, UK; d Laboratory for Social and Neural Systems Research (SNS), University of Zurich, Switzerland; e Newcastle University Medical School, Newcastle, UK

**Keywords:** Auditory evoked response, Magnetoencephalography, MEG, Auditory cortex, Attention, Attentional load, Change detection, Edge detection, Scene analysis

## Abstract

This study investigates how acoustic change-events are represented in a listener's brain when attention is strongly focused elsewhere. Using magneto-encephalography (MEG) we examine whether cortical responses to different kinds of changes in stimulus statistics are similarly influenced by attentional load, and whether the processing of such acoustic changes in auditory cortex depends on modality-specific or general processing resources. We investigated these issues by examining cortical responses to two basic forms of acoustic transitions: (1) Violations of a simple acoustic pattern and (2) the emergence of a regular pattern from a random one. To simulate a complex sensory environment, these patterns were presented concurrently with streams of auditory and visual decoys. Listeners were required to perform tasks of high- and low-attentional-load in these domains. Results demonstrate that while auditory attentional-load does not influence the cortical representation of simple violations of regularity, it significantly reduces the magnitude of responses to the emergence of a regular acoustic pattern, suggesting a fundamentally skewed representation of the unattended auditory scene. In contrast, visual attentional-load had no effect on either transition response, consistent with the hypothesis that processing resources necessary for change detection are modality-specific.

## Highlights

► We investigate MEG responses to acoustic changes when attention is focused away. ► Two change-types were studied: violation and emergence of a regular pattern. ► Auditory attentional-load reduces brain responses to emergence of regularity. ► However, responses to violation of regularity are not affected. ► Visual attentional-load has no effect on either transition response.

## Introduction

The ability to detect and respond quickly to changes in our surroundings – such as the appearance, disappearance or movement of an object – is critical to survival. Hearing plays a major role in this process by serving as an ‘early warning device', rapidly directing attention to new objects and events in the environment, and monitoring sources beyond the field of vision, in the dark, or in visually-cluttered surroundings. Indeed, we often *hear* changes in the environment before we *see* them.

The present study investigates whether the neural processes that sub-serve auditory change detection are automatic or depend on attentional resources — a question that is critical to understanding the fidelity of our pre-conscious representation of the ‘state of the world’, i.e. those aspects of the non-attended acoustic environment monitored by the brain while listeners' attention is focused elsewhere in the scene (Fritz et al., 2007).

The early, automatic, auditory change detection system has been traditionally studied with the mismatch-negativity (MMN) paradigm (Garrido et al., 2009; Näätänen et al., 1978; Polich, 2003). The MMN, a response generated by infrequent ‘deviant’ events embedded in a stream of repeating standard events, is hypothesized to reflect a discrepancy between the memory trace, or expectations, generated by the standard stimulus, and the new, deviant information (Winkler, 2007; Winkler et al., 1996). The degree to which MMN responses are dependent on attention has been a focus of investigation for several decades (e.g. Näätänen, 1992). It is widely asserted that attention directed beyond the auditory modality does not influence passively-elicited MMN responses (e.g. Alho et al., 1992; Muller-Gass et al., 2006, 2007; Restuccia et al., 2005; SanMiguel et al., 2008; Sculthorpe et al., 2008; Woods et al., 1992; but see Zhang et al., 2006), indicating modality-specific processing and generation of the MMN. Within the auditory modality, some reports have suggested that MMN responses are attenuated when attention is strongly focused on a competing sound stream (Alain and Izenberg, 2003; Woldorff and Hillyard, 1991). However, these data might be explained as a ‘competition’ effect resulting from the presence of identical deviants in both the attended and ignored auditory streams (Sussman et al., 2003). Tasks that eliminate this factor usually demonstrate no effect of attention on the MMN (e.g. Bendixen and Schröger, 2008; Näätänen et al., 2007; Sussman, 2007), resulting in the commonly held view that early auditory cortical mechanisms responsible for detecting changes in the auditory scene are generally independent of attention.

However, all previous studies on the role of attention for the MMN have focused exclusively on one kind of change — violation of regularity, i.e. a change that is manifested by the arrival of a signal that violates a previously established regular pattern. The majority of published work measured MMN responses using a fixed ‘standard’ stimulus and a ‘deviant’ that differs from the standard on some acoustic dimension (e.g. Müller et al., 2002; Muller-Gass et al., 2006; Restuccia et al., 2005; SanMiguel et al., 2008). More recently, the effect of attention has been measured for MMN responses elicited by violations of more complex patterns, such as an alternating pattern of two tones (ABAB…; Sculthorpe et al., 2008), or regularities defined by the frequency relation between successive tones (ascending vs. descending; Bendixen and Schröger, 2008). While changes, evidenced as a violation of a previously-acquired regularity, are commonly encountered by listeners (for example when a source embedded in the scene disappears, or changes its fluctuation properties), there exists another type of change, equally ubiquitous in natural scenes, that consists of the *emergence* of a regular pattern out of random fluctuation (as when an acoustic source appears from an ongoing random background). These kinds of changes have been much less explored (Wolff and Schröger, 2001; Horváth and Winkler, 2004; See also Bendixen et al., 2007) and, to our knowledge, no previous work has examined how processing of such changes depends on attentional resources.

**Fig. 1:**
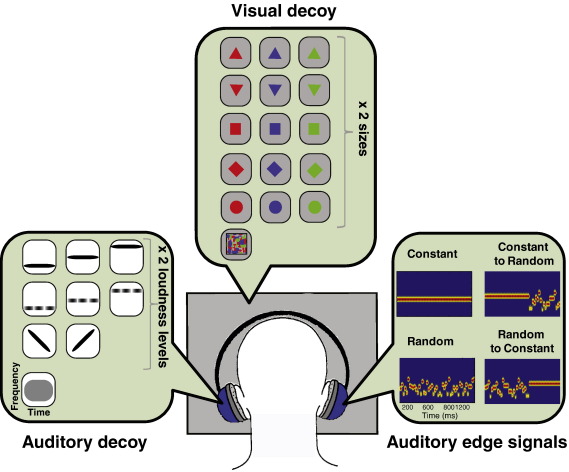
Schematic representation of the experimental set-up. Three streams of stimuli, ‘*auditory edge signals*’, ‘*auditory decoy signals*’ and ‘*visual decoy signals*’ were presented simultaneously to the participant. Attentional load was manipulated by instructing subjects to selectively attend to the auditory or visual decoy streams and perform a high- or low-attentional load task. Auditory edge signals were always ignored.

To model ‘violation of regularity’ and ‘emergence of regularity’ changes we use stimuli that contain a step change (‘temporal edge’) in the ongoing pattern of fluctuation (Fig. 1, right; Chait et al., 2007a, 2008). These stimuli are constructed as a sequence of tone pips with constant frequency that changes to a sequence of pips of random frequencies (constant to random, or ‘CR’ stimulus) or a sequence of tone pips of random frequency that changes to a sequence of pips of constant frequency (‘RC’ stimulus). These signals are conceptually similar to MMN step-change paradigms (e.g. Bendixen et al., 2007; Costa-Faidella et al., 2011) except that we use them to examine responses to the transitions between different sequences (rather than subtracting responses of deviants from standards). More importantly, these on-going stimuli also allow measurement of the responses to different (statistically diverse) acoustic transitions within a single experimental block.

While physically symmetric, RC and CR transitions are fundamentally different in nature. In the case of a CR transition, an observer can detect the event immediately as a violation of the current regularity. The opposite transition – RC – necessarily takes longer to detect because the observer must sample a sufficiently long epoch of the stimulus to distinguish the onset of regularity (repeating frequency) from a chance pattern within the on-going random sequence. We have previously demonstrated different neural processing for these transitions (Chait et al., 2007a, 2008) consistent with the theoretical argument that the two transitions require fundamentally different kinds of computations.

Here we ask two questions: (1) Is the detection of different forms of acoustic transitions similarly influenced by attentional load? Ostensibly, detecting violations of regularity might be computationally less expensive than detecting the emergence of a regular pattern and may therefore be less influenced by attentional load. (2) Does this process depend on modality-specific or modality-general computational resources?

To simulate a complex, multi-sensory environment and varying degrees of attentional involvement, ‘auditory edge’ stimuli were presented concurrently with streams of auditory and visual decoys. The form of the sensory input was identical in all conditions, and attentional load was manipulated by instructing participants to attend to either auditory or visual decoy streams, while performing a high- or low-attentional load task. Importantly, the auditory edge stream (the focus of this experiment) was always task-irrelevant. The pattern of auditory cortical responses to transitions in the auditory-edge stimuli were assessed in the context of the tasks performed by the listeners in order to determine whether and how auditory cortical sensitivity to (ignored) temporal-edges is affected by limited availability of processing resources.

## Experimental methods

### Participants

Nineteen subjects participated in the experiment. Data for two participants were excluded due to excessive magnetic artifacts. The mean age of the 17 remaining subjects (8 females) was 26.6 years. All but one were right handed (Oldfield, 1971), all reported normal hearing, normal or corrected-to-normal vision, and had no history of neurological disorders. Experimental procedures were approved by the research ethics committee of University College London, and written informed consent was obtained from each participant. Subjects were paid for their participation.

Due to a hardware malfunction, a significant number of button presses were not registered for the first 5 participants. The behavioral performance measures reported here are therefore based on the data from 12 subjects.

### Stimuli

Fig. 1 schematizes the experimental paradigm. Three streams of stimuli were presented concurrently, two auditory (in opposite ears) and one visual. Stimulation was identical in all conditions, but the behavioral task differed between conditions. MEG responses were analyzed relative to one auditory stream (‘auditory edge stimuli’) while the task involved either the other auditory stream (‘auditory decoys’) or the visual stream (‘visual decoys’), as detailed below.

‘*Auditory edge stimuli*’ consisted of a train of 30-ms tone pips (0 ms inter-tone interval) presented for a total duration of 1440 ms. Tone frequencies were drawn from a set of 20 values equally spaced on a logarithmic scale between 222 and 2000 Hz. The amplitude of each pip was shaped by initial and final 5 ms raised-cosine ramps. Four patterns of frequencies were presented: C (‘constant’), R (‘random’), CR (‘constant to random’) and RC (‘random to constant’). The C stimulus consisted of a sequence of tone pips of a constant frequency, the R stimulus of a sequence of pips with frequencies drawn randomly from the set of 20 values, the CR stimulus of an initial 840-ms constant frequency sequence followed by a 600-ms random sequence, and the RC stimulus of an initial 840-ms random sequence followed by a 600-ms constant frequency sequence. The C and R patterns served as controls for the RC and CR transitions that were the primary focus of the study (see Fig. 3) and also made the occurrence of transitions unpredictable. This was important since predictability of transitions seems to modify auditory cortical responses to edges (Chait et al., 2007b).

Forty signals were generated for each of the 4 patterns (C, R, CR, RC). CR and RC stimuli were created as mirror images of each other and trimmed to the required duration. Frequencies were drawn randomly from the above frequency set with the constraint that the change in frequency at the transition (at 840 ms post onset) was at least 20% in order to make it sufficiently salient. In a random sequence, it could happen by chance that two consecutive pips shared the same frequency: this occurred with a rate of about 5%.

The stimuli were created off-line and saved in 16-bit stereo wave format at a sampling rate of 44 kHz. They were presented to the listeners in a random order with an inter stimulus interval (ISI) randomized between 500 and 1000 ms.

‘*Visual decoy’ stimuli* consisted of five different shapes (circle, square, triangle, upside–down triangle and diamond) drawn in one of three colors (red, green or blue) and two sizes (visual angle of 7.4 or 2.86°), as well as a ‘random’ shape which was presented in only one size (visual angle of 13.7°). The ‘random’ shape stimulus consisted of a visual checker pattern, generated by multiple random-sized ellipses that were each drawn in a random color (red, green, or blue), and at a random position within the target square (see Fig. 1 for an example). Stimuli were presented in the center of a gray screen (RGB: 190,190,190) at a distance of about 52 cm from the subject's eyes. Overall, 31 different visual stimuli were presented with an inter-onset interval randomized between 200 and 300 ms and with zero ISI.

‘*Auditory decoy’ stimuli* consisted of nine different ‘auditory shapes’: (1) 200 Hz pure tone (2) 400 Hz pure tone (3) 800 Hz pure tone (4) 300 Hz pure tone amplitude modulated at 20 Hz (5) 600 Hz pure tone amplitude modulated at 20 Hz (6) 1200 Hz pure tone amplitude modulated at 20 Hz (7) Downwards FM glide from 800 to 400 Hz (8) upwards FM glide from 400 to 800 Hz (9) wide-band noise. Stimuli were 250 ms in duration and shaped by initial and final 25 ms raised-cosine ramps. All stimuli, except for the white noise, had a soft and louder version (24 dB difference). Overall, 17 different ‘auditory decoy’ stimuli were created off-line and saved in 16-bit stereo wave format at a sampling rate of 44 kHz. These stimuli were presented in a random order with ISI randomized between 150 and 350 ms.

The computer that controlled the presentation of the ‘auditory edge’ signals was different from the one that controlled the presentation of the decoy stimuli, to assure that stimulus sequences were not synchronized.

#### Paradigm

Participants sat in a darkened magnetically shielded room. The visual signals were presented on a screen, placed approximately half a meter in front of the subjects' eyes. Auditory signals were delivered dichotically to the subjects' ears (‘auditory edge’ signals to one ear and ‘auditory decoy’ to the other) with tubephones (E-A-RTONE 3A 10 Ω, Etymotic Research, Inc) inserted into the ear-canal. The level of the ‘auditory edge’ signals was 6 dB higher than the ‘auditory decoy’ signals. The overall stimulus level was adjusted, for each subject, to a comfortable listening level.

The experimental session was divided into blocks of about 6 min during which all three stimulus streams were present concurrently. The task (modality and difficulty) was constant within a block, but varied between blocks. Before the beginning of a block a message appeared on the screen instructing subjects to attend to the ‘visual decoy’ or ‘auditory decoy’ stimuli. The block was divided into 30 s long trials. At the beginning of a trial, the subject was briefly presented with a target in the attended modality, and had to memorize it and subsequently detect its occurrence during the trial, by pressing a button held in their right hand (rapid serial search paradigm). The target could occur between 0 and 3 times during a trial. The instructions encouraged speed and accuracy. Hits were defined as responses falling within a 1000 ms time window from a target. At the end of the trial the numbers of misses and false positives were briefly presented to the subject, immediately followed by the next trial, with a new target to memorize. The ear of presentation for ‘auditory edge’ and ‘auditory decoy’ stimuli was counter-balanced across blocks.

For each modality, the task was either easy (‘low attentional load’) or hard (‘high attentional load’). In auditory low-load blocks, the target to be detected was the same in each trial and was always the noise stimulus. This stimulus is physically very different from the rest (wide band vs. narrow band) and was easily detected within the stimulus stream. In auditory high-load blocks the target could be any of the other signals, and changed from trial to trial. Subjects had to memorize correctly both the signal ‘shape’ and loudness. Similarly, in visual low-load blocks the target was always the multi-colored random shape stimulus and in high-load blocks subject had to memorize a different shape, color and size combination in each trial. In sum, 8 different blocks (2 modalities × 2 loads × counter balanced ear of presentation for auditory signals) were presented in random order. To ensure that sensory stimulation was identical across blocks, subjects were instructed to fixate at the center of the screen at all times and this was verified with eye tracking (iView X, SMI, Germany). Between blocks, subjects were permitted a short rest but were required to stay still.

An important aspect of our decoy tasks is the fast stimulus presentation rate (Muller-Gass et al., 2006; Otten et al., 2000). When decoy stimuli are presented at a fast rate attentional switches between the attended and ignored streams are unlikely. Indeed, when questioned at the end of the experiment, subjects mostly reported not noticing that the ignored ‘auditory edge’ stream included transitions within stimuli.

### Procedure

The experimental session included three phases: First, for approximately 15 min, subjects practiced the tasks (*in situ*). Recording sessions then began with a preliminary functional source-localizer recording, followed by the main experiment (8 blocks). In the functional source-localizer recording subjects listened to 200 repetitions of a 1 kHz 50 ms sinusoidal tone (ISI randomized between 750 and 1550 ms). These responses were used to verify that the subject was positioned properly in the machine, that signals from auditory cortex had a satisfactory signal to noise ratio (SNR), and to determine which MEG channels best responded to activity within auditory cortex.

### Neuromagnetic recording and data analysis

The magnetic signals were recorded using a CTF-275 MEG system (axial gradiometers, 275 channels, 30 reference channels; VSMMedTech, Canda). Data were acquired continuously with a sampling rate of 300 Hz and a 100 Hz hardware low pass filter. Offline, the data were noise-reduced using the Time-Shift Principle Component Analysis algorithm (TSPCA; de Cheveigné and Simon, 2007) and then low pass filtered (zero-phase Butterworth filter) at 30 Hz.

Functional localizer data were divided into 700 ms epochs, including 200 ms pre-onset, and baseline-corrected to the pre-onset interval. The M100 onset response (Hari, 1990; Roberts et al., 2000) was identified for each subject as a dipole-like pattern (i.e. a source/sink pair) in the magnetic field contour plots distributed over the temporal region of each hemisphere. The M100 current source is quite robustly localized to the upper banks of the superior temporal gyrus in both hemispheres (Hari, 1990; Lütkenhöner and Steinsträter, 1998). For each subject, the 40 strongest channels at the peak of the M100 (20 in each hemisphere) were considered to best reflect activity in the auditory cortex and thus chosen for the analysis of the experimental data. This procedure serves the dual purpose of enhancing the auditory response components over other response components, and compensating for any channel-misalignment between subjects.

For the main experiment data, 2200 ms epochs (including 200 ms pre onset) were created for each of the stimulus conditions (2 (vis/aud) × 2 (high/low attentional load) × 4 auditory edge stimuli (C, R, CR, RC)), resulting in 80 epochs per condition. Epochs with amplitudes larger than 3 pT (~ 3%), such as what would be caused by eye blinks, were considered artifactual and discarded. Additionally, epochs during which subjects were not fixating in the center of the screen (< 1%) were excluded from analysis. The rest were averaged. In each hemisphere, the root mean square (RMS) of the field strength across the 20 channels, selected in the functional source-localizer run, was calculated for each sample point. Thirty-two RMS time series (16 conditions × two hemispheres) were thus created for each subject.

The time course of the RMS, reflecting instantaneous amplitude of neural responses, was used as a measure of the dynamics of brain responses. The congruence of activation time course across subjects was evaluated using the bootstrap method (Efron and Tibshirani, 1993; 1000 iterations; balanced) based on the individual RMS time series as described in Chait et al. (2007a). For illustration purposes, we plot a group-RMS (RMS of individual subject RMSs), but statistical analysis was always performed over subjects, independently for each hemisphere.

To compare the activation between conditions, we used a repeated-measures analysis in which, for each subject, the squared RMS value of one condition was subtracted from the squared RMS value of the other condition, and the 17 individual difference time-series were subjected to a bootstrap analysis (1000 iterations; balanced; Efron and Tibshirani, 1993). At each time point, the proportion of iterations below the zero line was counted. If that proportion was less than 1%, or more than 99% for 8 adjacent samples, the difference was judged to be significant. This figure (8 samples) was determined based on a permutation analysis designed to measure the ‘false discovery rate’. In brief, we ran an iterative analysis (1000 repetitions) to simulate the H0 hypothesis (no difference between conditions), where instead of the actual data time-series we used random time-series (created by permuting the samples in the original data; to simulate the level of temporal coherence introduced by the low-pass filter, the permuted time-series underwent the same pre-processing procedures as for the true data). Using the repeated-measure analysis described above, the maximum number of consecutive samples that fell outside of the 1%/99% criterion was computed. The longest such sequence was of length 7 (and occurred roughly 1% of the time) and, therefore, only sequences of 8 samples or longer were considered statistically significant.

Peak latencies and amplitudes were measured by selecting, for each subject and condition, the maximum (or minimum) value within the relevant time window, defined as ± 20 ms centered around the group-RMS peak. These data were submitted to a repeated-measures ANOVA. The α level was set, a-priori, to 0.05. The Greenhouse–Geisser correction was applied where appropriate.

## Results

### Behavioral data

**Fig. 2:**
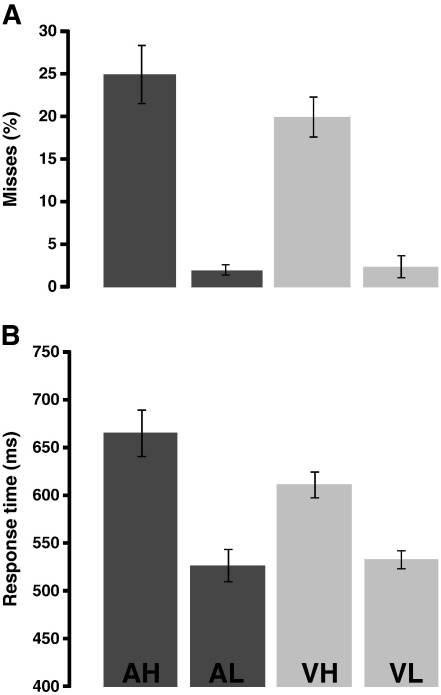
Behavioral performance. AH: Auditory high load; AL: Auditory low load; VH: Visual high load; VL: Visual low load. A: Miss rates. B: Response times. Error bars are one standard error of the mean.

Subjects had little difficulty performing auditory and visual tasks requiring low attentional load, resulting in ceiling performance in both cases (Fig. 2A; mean miss rate of 2.1% and 2.5%, respectively). However, performance was significantly reduced for tasks requiring high attentional load (mean miss rate of 25.6% and 19.1%, respectively). A repeated-measures ANOVA with factors ‘modality’ and ‘load’ showed a significant main effect for ‘load’ only (*p* < 0.001), and no interaction, confirming that our choice of tasks was effective in manipulating attentional load to a similar degree for both sensory domains. False positive rates were small, but subjects generated more false positives in the high-load auditory task (3%) than the high-load visual task (0.5%).

Response times (RTs) in low-load tasks were similar for both modalities (auditory: 534.12 ms; visual: 540.3 ms), and were significantly longer for high-load tasks (auditory: 676.5 ms; visual: 617.8 ms). A repeated-measures ANOVA with ‘modality’ and ‘load’ as factors revealed significant main effects of load (*p* = 0.012) and modality (*p* < 0.001) as well as an interaction (*p* = 0.019). This was due to the fact that RTs in the high-load auditory task were slower (by about 60 ms) than in the high-load visual task, presumably because the features distinguishing auditory decoy stimuli related to temporally evolving properties whose extraction required longer exposure to the stimulus. Importantly, for purposes of the present study, both the RT and accuracy data confirmed that the attentional load manipulation was effective in influencing behavioral performance in both the visual and the auditory tasks, with comparable detection levels in both domains.

### MEG data

#### MEG responses to auditory edge stimuli

**Fig. 3:**
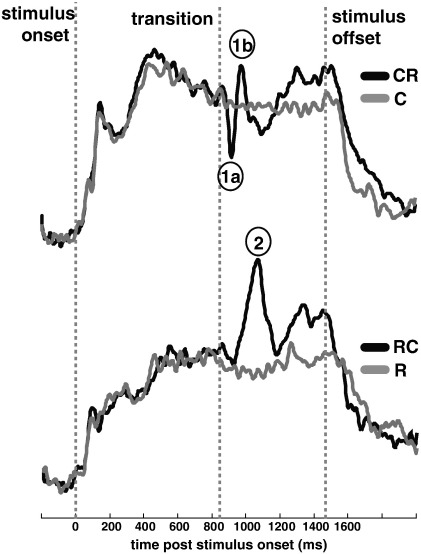
Group-RMS (RMS of individual subject RMSs) of right hemisphere auditory cortical responses evoked by auditory edge stimuli (top: constant-to-random, CR; bottom: random to constant, RC) collapsed over attentional load conditions (left hemisphere responses are comparable). Change stimuli (CR and RC) in black and their respective control (no change) conditions in gray. The origin of the time scale coincides with the onset of the signals and the transition occurs at 840 ms post stimulus onset. Transition responses exhibit temporal/morphological differences between conditions. ‘1a’, ‘1b’ and ‘2’ tag the prominent deflections in each transition.

To demonstrate the general response pattern to the two forms of transition, MEG activity was first assessed across all attentional load conditions (Fig. 3). Plotted are group root-mean-square (group-RMS; RMS of individual subject RMSs) of auditory-evoked responses to constant-to-random (CR) and random-to-constant (RC) transitions and the control conditions (C and R, respectively). Responses to C and R tone sequences show a similar pattern – an onset response (peaking about 100 ms post stimulus onset) followed by a rise to a sustained response – maintained for the duration of the stimulus, and a return to baseline following stimulus offset. The dynamics of the response to the transition, however, differ substantially for the CR and RC conditions. Upon transition from a sequence of constant to random tone pips, the MEG signal shows a sharp drop in the sustained response (‘1a’ in Fig. 3), reaching a minimum some 70-ms following the transition (‘M50’ response). A repeated-measures bootstrap (see Experimental methods section) indicates that the initial difference in MEG responses between the CR stimulus and its control (C) stimulus emerges some 60-ms post transition in the right hemisphere, and 63-ms post transition in the left. This initial deflection (‘1a’ in Fig. 3) is then followed by a peak, ‘1b’ in Fig. 3, with a latency of 136-ms post transition in the right hemisphere and 177 ms in the left. Together, these peaks are reminiscent of the MEG M50–M100 response complex (P1–N1 in the electroencephalogram) commonly evoked by stimulus onset or transitions (e.g. Krumbholz et al., 2003; Martin and Boothroyd, 2000; Ritter et al., 2005; Ross et al., 2004; Yamashiro et al., 2011).

In response to the opposite transition – from random to constant frequency – MEG activity peaks much later, about 200 ms following the transition (‘2’ in Fig. 3) and with a very different shape (increase in magnetic field strength) to that evoked by the CR transition. A repeated-measures bootstrap indicates that the first difference from the control (no change stimuli) emerges 126 ms after the transition in the right hemisphere and 140 ms post-transition in the left. The striking absence of a M50 response here reveals a functional dissociation between the transition-evoked M50 and M100 responses. Cortical detection of the RC transition is therefore not only delayed with respect to the CR transition, but also involves a different sequence of MEG deflections.

These findings replicate previous studies which demonstrated different responses for the two kinds of temporal edges (Chait et al., 2007a, 2008), but now for conditions in which attention was directed *away* from the edge stimuli.

#### Effects of attentional load

To assess the effect of attentional load on responses to temporal edges, the data were examined in 4 conditions: auditory low load (AL), auditory high load (AH), visual low load (VL) and visual high load (VH). Each condition represented data from 80 trials for each of the C, R, CR, RC stimuli.

#### Attention directed to the auditory modality

For blocks in which attention was focused on the auditory decoys, a significant reduction in the amplitude of response to RC transitions was observed for the high-load (AH) condition. Panel 4E shows average peak amplitudes for the three deflections marked in Fig. 3. A repeated-measures ANOVA with ‘deflection’ (‘1a’, ‘1b’, ‘2’; see Fig. 3), ‘load’, and ‘hemisphere’ as factors showed a trivial main effect of ‘deflection’ (due to the different absolute amplitudes of each peak) and a significant interaction of ‘deflection’ × ‘load’ (*p* = 0.007). *Post hoc* analysis revealed that only the random-to-constant (RC) transition was significantly influenced by attentional load (*p* = 0.005; for other deflections *p* > 0.24), with a reduction in MEG amplitude of around 20% compared to that for the low-load condition. There was no influence of attentional load on the latency of the various deflections.

**Fig. 4:**
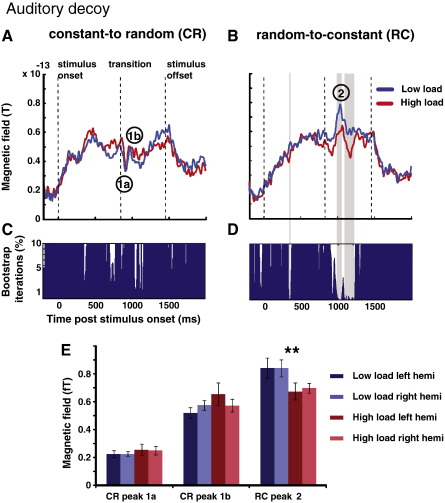
The effect of varying the attentional load in the auditory decoy task on edge detection responses in auditory cortex. A: CR transition. Group-RMS of right hemisphere auditory cortical evoked responses in the low load (blue) and high load (red) conditions. B: RC transition Group-RMS of right hemisphere auditory cortical evoked responses in the low load (blue) and high load (red) condition. Shaded areas mark time intervals where a significant difference is found between load conditions. B,D Repeated measures bootstrap analysis. For each time point, we plot the minimum percentage (capped at 10% for clarity) of bootstrap iteration located above or below zero. For a difference to be judged as significant this number must not exceed 1% (see Experimental methods section for additional constraints). E peak amplitudes of the two main CR peaks (‘1a’ and ‘1b’) and the RC peak (‘2’) in both hemispheres under the different load conditions. Load had a significant effect on the amplitude of the RC response only.

In addition to this temporal ‘region of interest’ analysis, a ‘blind’ method was used to explore the extent to which these intervals emerge from the data: Figs. 4A and B show the group-RMS of the auditory evoked responses to CR and RC edges, respectively. Gray shading indicates the temporal intervals where a repeated-measures bootstrap indicated a significant difference between low- and high-load conditions. Whereas no significant differences appear in the CR stimuli between attentional conditions, a significant decrease in the amplitude of the RC transition response is observed in the high-load task. To demonstrate the significance of the effects, panels 4C and 4D display the results of the repeated measures bootstrap analysis. For each point in time, the minimum ratio (capped at 10% for clarity) of bootstrap iterations located above or below zero is plotted. For a difference to be judged as significant, this number has to be < 1% (99% of the iterations have to lie on one side of the zero line; see Experimental methods section for additional constraints). Such effects were clearly evident for RC transitions (Fig. 4D), but not for CR transitions (Fig. 4C).

While a slow-wave shift appears to occur prior to the transition in the RC stimuli (Fig. 4B), the bootstrap analysis (Fig. 4D) indicates that this effect is not statistically significant, and that the difference between the high and low load curves emerges only at around 980-ms post onset (140-ms post transition) and is restricted to the interval around the response peak. An additional analysis (see Supplementary Fig. 1A), involving base-line correction of the response relative to the pre-transition interval, resulted in an essentially identical significance pattern. This confirms that the effect is restricted to the response peak and is not due to a baseline shift. Additionally, load had no significant effect on responses to the control (no transition; C or R) stimuli (Supplementary Fig. 1B), further suggesting that the effects seen in Fig. 4 are specific to transition responses and are not due to a baseline shift in the R stimulus.

#### Attention directed to the visual modality

**Fig. 5:**
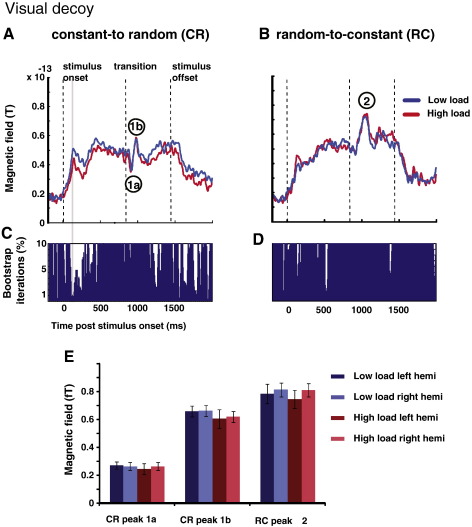
The effect of varying the attentional load in the visual decoy task on edge detection responses in auditory cortex. A: CR transition. Group-RMS of right hemisphere auditory cortical evoked responses in the low load (blue) and high load (red) condition. B: RC transition Group-RMS of right hemisphere auditory cortical evoked responses in the low load (blue) and high load (red) condition. Shaded areas mark time intervals where a significant difference is found between load conditions. B,D Repeated measures bootstrap analysis. For each time point, we plot the minimum percentage (capped at 10% for clarity) of bootstrap iteration located above or below zero. For a difference to be judged as significant this number must not exceed 1% (see Experimental methods section for additional constraints). E peak amplitudes of the two main CR peaks (‘1a’ and ‘1b’) and the RC peak (‘2’) in both hemispheres under the different load conditions. Load had no significant effect on either RC or CR responses.

In contrast to auditory attentional load, visual attentional load had no effect on the MEG response (Fig. 5). A repeated measures bootstrap indicated no significant difference between low- and high-load conditions in either transition (panels 5C and 5D). Panel 5E shows average peak latencies for the three deflections marked in Fig. 3. A repeated measures ANOVA with ‘deflection’ (‘1a’, ‘1b’, ‘2’), ‘load’, and ‘hemisphere’ as factors, revealed no significant effects (for all *p* > 0.19).

Consequently, these data reveal a fundamental difference in the effects of attentional load in the visual and auditory domain on cortical processing of unattended auditory temporal edges. This difference was confirmed statistically by means of a repeated-measures ANOVA with ‘modality’, ‘load’, ‘deflection’ and ‘hemisphere’ as factors, which revealed a significant interaction of ‘modality’ × ‘load’ × ‘deflection’ (*p* = 0.006), again demonstrating that the effect of attentional load is found only for the RC deflection under conditions of high auditory attentional load.

The data demonstrate that the amplitude differences observed at the response peaks are probably not attributable to differences in sustained responses which preceded the transition. Furthermore, while the low- and high-load tasks differed in the number of executed button presses, this also cannot be the source of the observed effect, as only RC transitions in the auditory high-load condition were affected. Indeed, any explanation of the effect in terms of certain deflections in the MEG signal being inherently more susceptible to task-related ‘brain noise’ must be discarded on the grounds that such an explanation would predict identical patterns in visual and auditory tasks.

#### Effect of attentional load on onset/sustained responses to auditory edge stimuli

As evident from Fig. 4 there is no effect of auditory attentional load on stimulus onset responses (0–200 ms post stimulus onset). The data in Fig. 5 show a possible effect of visual load at the onset of C but not R signals, but since this onset effect is restricted to visual load conditions it is difficult to interpret. In neither modality does attentional load appear to influence sustained responses (300–840 ms post stimulus onset; see also Muller-Gass et al., 2006).

## Discussion

The present study examined the effect of attentional load on cortical processing of acoustic transitions in which the sound pattern changed from a constant-frequency to a random-frequency sequence, or *vice versa*. The major finding is that auditory cortical responses to the emergence of a repeating pattern, but not to violations of this regularity, are influenced by the level of attentional load required to perform a competing auditory task. This observation suggests that the mechanisms for detecting the emergence or violation of such a simple regularity in the auditory domain are not equally automatic. Notably, these effects are limited to conditions where attention is directed to the auditory modality; a structurally similar competing visual task had no effect on cortical processing of either form of acoustic transition, underlining the dependence of these operations on modality-specific attentional resources.

### Auditory cortical responses to CR and RC transitions

As has been shown previously (Chait et al., 2007a, 2007b, 2008), CR and RC transitions evoke different sequences of brain responses. A transition from a sequence of constant to random tone pips, gives rise to a M50–M100-like response complex, whereas responses to the opposite transition, from random to constant frequency, peak much later and are dominated by a late M100-like response without a preceding M50 deflection. Previous studies have observed an essentially-identical MEG response profile for a variety of signals that differed physically but nevertheless shared the abstract property of transitions from – or to – a predictable pattern. For example, transitions between regular and random frequency patterns (Chait et al., 2007a, 2008), transitions between regular and random patterns in the dimension of inter-aural correlation (Chait et al., 2005), transitions between random noise, and ‘regular’ iterated noise (Rupp et al., 2005) or between regular and random click trains (Gutschalk et al., 2004). This suggests that the observed dynamics are not specific to transitions in any particular acoustic feature, but rather depend on the statistical patterns (regular to irregular, irregular to regular) common to these stimuli.

Wolff and Schröger (2001) and Horváth and Winkler (2004; see also Näätänen and Rinne, 2002; Ritter et al., 1992) examined responses to occasionally repeating sounds in a sequence of random frequency tones, comparable to the RC stimulus here, and reported the presence of an MMN response, time locked to the second repeating tone. However, while the MMN response obtained in those studies resembled a standard oddball MMN (MMN to a violation of regularity), the present paradigm reveals fundamental differences between responses to CR and RC transitions that are not usually reported for MMN responses (Chait et al., 2008; see also Grimm et al., 2011) . Thus, while it is likely that some of the neural processes contributing to the responses we observe overlap with those underlying the MMN, in certain cases the present ‘transition-response’ paradigm allows a more refined view of the different processes at work during auditory change detection.

The differences we observe between CR and RC transitions contradict a simple default hypothesis that all transitions are processed by the same underlying neural hardware, in which case we would expect the same pattern of responses to both types of transitions distinguishable only by a latency difference. Instead, our data suggest, in line with previous results, that at least partially separate neural computations are employed in the process of auditory temporal edge detection, depending on the type of transition encountered (see also Chait et al., 2008). The current study demonstrates that the two mechanisms are differentially affected by attention, which further strengthens our theoretical synthesis based on distinct underlying neural mechanisms.

### Auditory attentional load effects

Our data suggest, in accordance with results from the MMN literature (e.g. Alho et al., 1992; Bendixen and Schröger, 2008; Muller-Gass et al., 2006, 2007; Näätänen et al., 2007; Restuccia et al., 2005; SanMiguel et al., 2008; Sculthorpe et al., 2008; Sussman, 2007; Woods et al., 1992), that sensitivity to violation-of-regularity edges (which are similar to the kinds of changes which have been previously studied with the classic oddball MMN paradigm), does not deteriorate with increasing auditory load. In contrast to these established findings, we find here that sensitivity to changes manifested as the emergence of structure from a random sequence (a type of change which has been relatively unexplored; see e.g. Bendixen et al., 2007) is significantly reduced when listeners' attention is strongly focused on a competing auditory task.

Indeed, while formally symmetric, RC and CR transitions are fundamentally different in nature. In the case of CR transitions, an observer can detect the event immediately as a violation of the current regularity. To detect the opposite transition – RC – the observer must sample a sufficiently-long epoch of the stimulus and compare past and present fluctuation statistics. The data thus suggest that regularity build-up requires substantial computational resources. However once a regularity model has been established, detection of deviations is automatic and independent of attention. This result represents a conceptual shift from the traditional approach, which so far did not differentiate changes according to their statistical properties. The present data indeed suggest that cortical processing of changes in the environment, beyond the focus of attention, critically depends on the statistical nature of those changes.

Assuming that the observed responses reflect mechanisms which pre-consciously encode external change events into some sort of internal representation of the ‘state of the world’, the finding that brain responses to different events that are equally un-attended (and equally salient) can nevertheless be differentially influenced by attentional load, suggests that strongly focused attention may lead to a skewed internal model of the (ignored) acoustic environment. That is to say, certain events would be represented with more fidelity than others, potentially leading to behavioral consequences (e.g. listeners being more distracted by CR- rather than RC-type events).

### Modality specificity of attentional load effects

A fundamental question in the study of attention concerns the relation of the processing resources engaged by each of the different senses. Are resources shared across a single, central pool accessible to all sensory modalities, or are specialized sensory resources reserved for each modality independently (e.g. Alais et al., 2006; Duncan et al., 1997; Larsen et al., 2003)? In the context of attentional load, this issue concerns the degree to which strongly focused attention affects the processing of *task-irrelevant* (‘to be ignored’) stimuli of the same or different modality as the attended stimulus stream (Lavie, 2005). Typically, studies which employ fast target-stream stimulation rates that reduce the chance of shifting attention to the to-be-ignored modality demonstrate that brain responses to task-irrelevant stimuli are independent of attentional load when attention is focused on a different modality (Dyson et al., 2005; Muller-Gass et al., 2005; Rees et al., 2001; Restuccia et al., 2005; Sculthorpe et al., 2008; Talsma et al., 2006). For example, Rees et al. (1997, 2001) showed that visual load, but not auditory load, modulates processing of a task-irrelevant visual motion stimulus. Talsma et al. (2006) reported that cortical responses elicited by a repeating visual-letter stream were significantly larger when concurrent auditory stimuli were attended than when other concurrent visual stimuli were attended. Likewise, numerous studies investigating the effect of visual load on auditory MMN responses report no influence of strongly focused visual attention on the detection of change in the auditory stimulus stream (Dyson et al., 2005; Müller et al., 2002; Muller-Gass et al., 2005; Restuccia et al., 2005; Sculthorpe et al., 2008).

The present study differs from these previous studies in two basic aspects. First, the effects of auditory and visual decoy tasks *simultaneously* were investigated, within the same recording session and using identical sensory stimulation in all conditions. This allowed the exclusion of confounding effects of stimulus differences, highlighting effects that can be ascribed specifically to the task. Second, as discussed above, the stimulus set contains more dimensions of change than those widely employed in the MMN paradigm, revealing that different types of changes (RC and CR) are differentially influenced by attentional load.

While it is difficult to exclude the possibility that other visual tasks with different stimuli or spatial arrangements might elicit a different set of results, it is worth noting that the null effect in the visual load condition appears convincing (Fig. 5), with no obvious trends. The data are therefore consistent with modality-specific effects of attentional load on the processing of acoustic changes, and suggest that mechanisms contributing to detection of the emergence of regularity rely on processing resources reserved exclusively for the auditory modality. Responses to RC transitions were attenuated only when attention prioritized processing of concurrent auditory signals, thereby depleting available processing resources in the auditory domain.

The following are the supplementary materials related to this article.

Supplementary Fig. 1 A: Re-analysis of the data in Fig. 4, base-corrected relative to the pre-transition interval (-50:0 relative to the transition time). Plotted are group-RMS of right hemisphere auditory cortical evoked responses in the low load (blue) and high load (red) conditions. Shaded areas mark time intervals where a significant difference is found between load conditions. Bottom panels show the repeated measures bootstrap analysis. For each time point, we plot the minimum percentage (capped at 10% for clarity) of bootstrap iteration located above or below zero. For a difference to be judged as significant this number must not exceed 1% (see Experimental methods section for additional constraints). The figure demonstrates that the effect of load on the amplitude of the RC transition survives this re-analysis, confirming that the difference between ‘high’ and ‘low’ load in the RC transition is restricted to the interval around the peak, and is likely not due to a baseline shift which precedes the transition. B: The effect of varying the attentional load in the auditory decoy task on responses to the control (no transition) stimuli. Plotted are group-RMS of right hemisphere auditory cortical evoked responses in the low load (blue) and high load (red) conditions. Shaded areas mark time intervals where a significant difference is found between load conditions. Bottom panels show the repeated measures bootstrap analysis. For each time point, we plot the minimum percentage (capped at 10% for clarity) of bootstrap iteration located above or below zero. For a difference to be judged as significant this number must not exceed 1% (see Experimental methods section for additional constraints). The figure demonstrates that load had no significant effect on either C or R responses, further suggesting that the effects seen in Fig. 4 are specific to transition responses and are not due to a baseline shift in the R stimulus.

Supplementary materials related to this article can be found online at doi:10.1016/j.neuroimage.2011.09.006.
