# Supplementary figures and images for: Cortical responses to changes in acoustic regularity are differentially modulated by attentional load
Source: Neuroimage. 2012 Jan 16;59(2-5):1932–41. doi: 10.1016/j.neuroimage.2011.09.006 (PMC3271381; doi:10.1016/j.neuroimage.2011.09.006)

## A pre-transition baseline

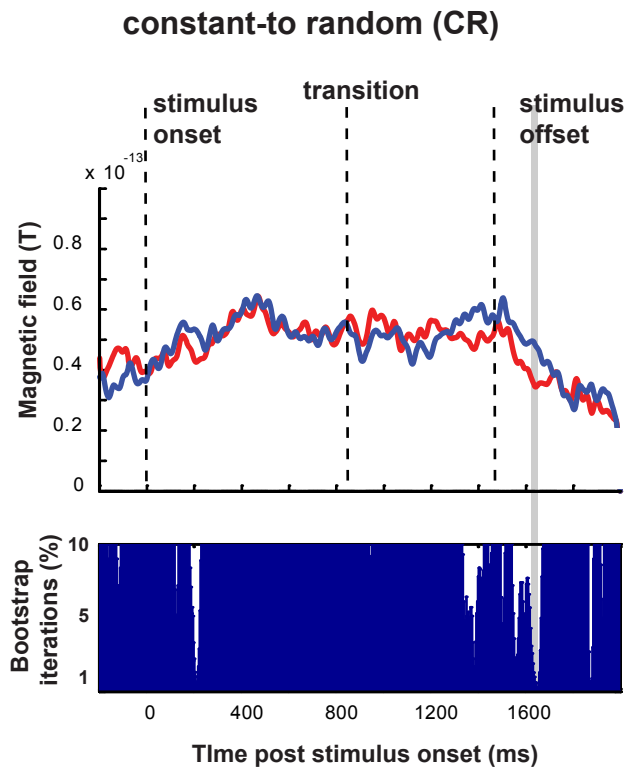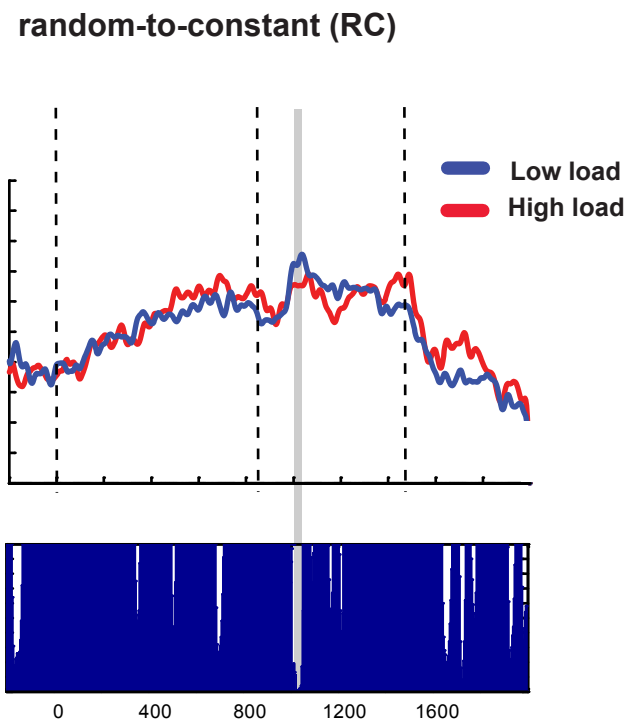

## B control (no transition) conditions

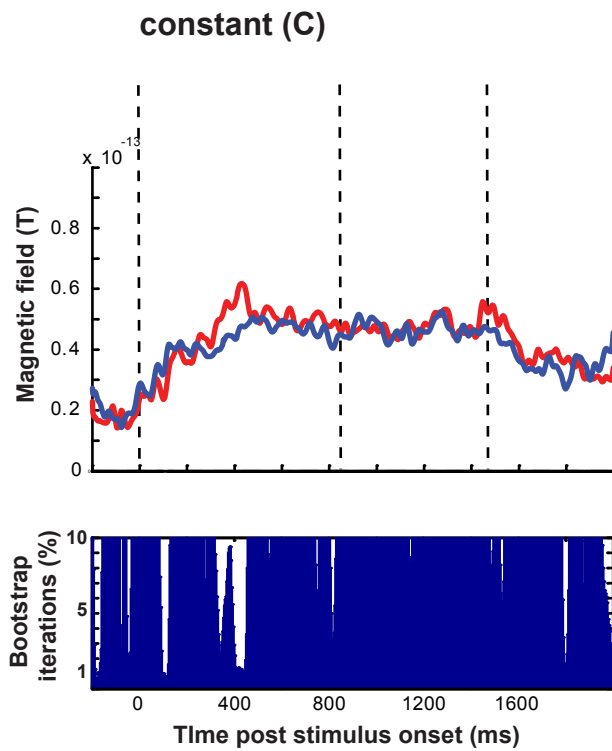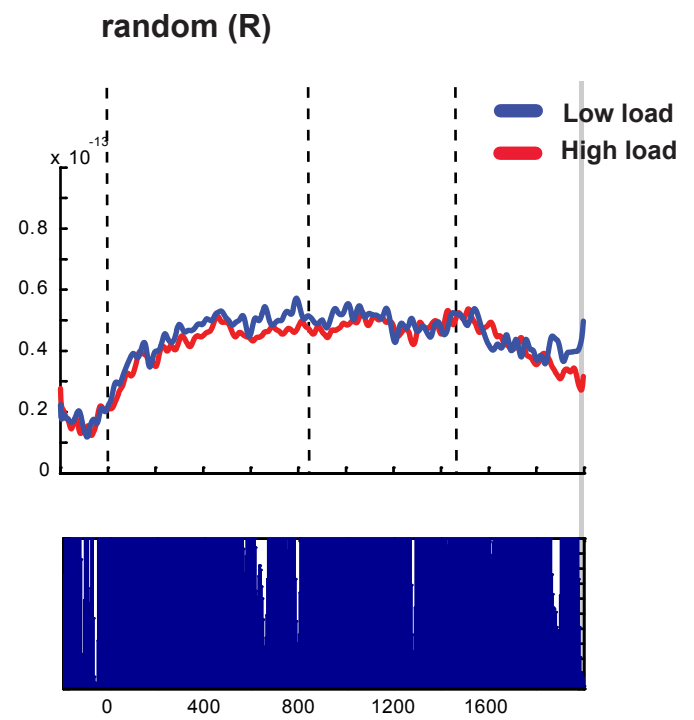

Supplement: Supplementary Fig. 1 — A: Re-analysis of the data in Fig. 4, base-corrected relative to the pre-transition interval (-50:0 relative to the transition time). Plotted are group-RMS of right hemisphere auditory cortical evoked responses in the low load (blue) and high load (red) conditions. Shaded areas mark time intervals where a significant difference is found between load conditions. Bottom panels show the repeated measures bootstrap analysis. For each time point, we plot the minimum percentage (capped at 10% for clarity) of bootstrap iteration located above or below zero. For a difference to be judged as significant this number must not exceed 1% (see Experimental methods section for additional constraints). The figure demonstrates that the effect of load on the amplitude of the RC transition survives this re-analysis, confirming that the difference between ‘high’ and ‘low’ load in the RC transition is restricted to the interval around the peak, and is likely not due to a baseline shift which precedes the transition. B: The effect of varying the attentional load in the auditory decoy task on responses to the control (no transition) stimuli. Plotted are group-RMS of right hemisphere auditory cortical evoked responses in the low load (blue) and high load (red) conditions. Shaded areas mark time intervals where a significant difference is found between load conditions. Bottom panels show the repeated measures bootstrap analysis. For each time point, we plot the minimum percentage (capped at 10% for clarity) of bootstrap iteration located above or below zero. For a difference to be judged as significant this number must not exceed 1% (see Experimental methods section for additional constraints). The figure demonstrates that load had no significant effect on either C or R responses, further suggesting that the effects seen in Fig. 4 are specific to transition responses and are not due to a baseline shift in the R stimulus. [file mmc1.pdf]
